# Gene Expression Profiles of Circular RNAs and MicroRNAs in Chronic Rhinosinusitis With Nasal Polyps

**DOI:** 10.3389/fmolb.2021.643504

**Published:** 2021-05-28

**Authors:** Jieqing Yu, Xue Kang, Yuanping Xiong, Qing Luo, Daofeng Dai, Jing Ye

**Affiliations:** ^1^ Department of Otorhinolaryngology Head and Neck Surgery, The First Affiliated Hospital of Nanchang University, Nanchang, China; ^2^ Jiangxi Otorhinolaryngology Head and Neck Surgery Institute, Nanchang, China; ^3^ Department of Otorhinolaryngology Head and Neck Surgery, Jiangxi Provincial Children’s Hospital, Nanchang, China

**Keywords:** chronic rhinosinusitis with nasal polyps, circular RNA, micro RNA, microarray analysis, gene express profile

## Abstract

**Introduction:** Chronic rhinosinusitis (CRS) is often classified primarily on the basis of the absence or presence of nasal polyps (NPs), that is, as CRS with nasal polyps (CRSwNP) or CRS without nasal polyps (CRSsNP). Additionally, according to the percentage of eosinophils, CRSwNP can be further divided into eosinophilic CRSwNP (ECRSwNP) and non-ECRSwNP. CRSwNP is a significant public health problem with a considerable socioeconomic burden. Previous research reported that the pathophysiology of CRSwNP is a complex, multifactorial disease. There have been many studies on its etiology, but its pathogenesis remains unclear. Dysregulated expression of microRNAs (miRNAs) has been shown in psoriasis, rheumatoid arthritis, pulmonary fibrosis, and allergic asthma. Circular RNAs (circRNAs) are also involved in inflammatory diseases such as rheumatoid arthritis, septic acute kidney injury, myocardial ischemia/reperfusion injury, and sepsis-induced liver damage. The function of miRNAs in various diseases, including CRSwNP, is a research hotspot. In contrast, there have been no studies on circRNAs in CRSwNP. Overall, little is known about the functions of circRNAs and miRNAs in CRSwNP. This study aimed to investigate the expression of circRNAs and miRNAs in a CRSwNP group and a control group to determine whether these molecules are related to the occurrence and development of CRSwNP.

**Methods:** Nine nasal mucosa samples were collected, namely, three ECRSwNP samples, three non-ECRSwNP samples, and three control samples, for genomic microarray analysis of circRNA and microRNA expression. All of the tissue samples were from patients who were undergoing functional endoscopic sinus surgery in our department. Then we selected some differentially expressed miRNAs and circRNAs for qPCR verification. Meanwhile, GO enrichment analysis and KEGG pathway analysis were applied to predict the biological functions of aberrantly expressed circRNAs and miRNAs based on the GO and KEGG databases. Receiver operating characteristic (ROC) curve analysis and principal component analysis (PCA) were performed to confirm these molecules are involved in the occurrence and development of CRSwNP.

**Results:** In total, 2,875 circRNAs showed significant differential expression in the CRSwNP group. Specifically, 1794 circRNAs were downregulated and 1,081 circRNAs were upregulated. In the CRSwNP group, the expression of 192 miRNAs was significantly downregulated, and none of the miRNAs were significantly upregulated. GO and KEGG analysis showed differential circRNAs and miRNAs were enriched in “amoebiasis,” “salivary secretion,” “pathways in cancer,” and “endocytosis.” Through qRT-PCR verification, the expression profiles of hsa-circ-0031593, hsa-circ-0031594, hsa-miR-132-3p, hsa-miR-145-5p, hsa-miR-146a-5p, and hsa-miR-27b-3p were shown to have statistical differences. In addition, ROC curve analysis showed that the molecules with the two highest AUCs were hsa-circ-0031593 with AUC 0.8353 and hsa-miR-145-5p with AUC 0.8690. Through PCA with the six ncRNAs, the first principal component explained variance ratio was 98.87%. The AUC of the six ncRNAs was 0.8657.

**Conclusion:** In our study, the expression profiles of ECRSwNP and non-ECRSwNP had no statistical differences. The differentially expressed circRNAs and miRNAs between CRSwNP and control may play important roles in the pathogenesis of CRSwNP. Altered expression of hsa-circ-0031593 and hsa-miR-145-5p have the strongest evidence for involvement in the occurrence and development of CRSwNP because their AUCs are higher than the other molecules tested in this study.

## Introduction

Chronic rhinosinusitis with nasal polyps (CRSwNP) is a significant public health problem with a considerable socioeconomic burden. Chronic rhinosinusitis (CRS), which is characterized by persistent mucosa inflammation of the sinuses, is one of the most common chronic diseases, and its pathophysiology remains unclear (AI-Sayed et al., 2017).

According to whether nasal polyps exist or not, CRS is often classified as CRSwNP or CRS without nasal polyps (CRSsNP) (Cho et al., 2017). CRSwNP remains a challenging clinical problem due to its propensity for recurrence. According to the percentage of eosinophils, CRSwNP can be classified as eosinophilic CRSwNP (ECRSwNP), with an eosinophil count ≥10%, and non-eosinophilic CRSwNP (non-ECRSwNP), with an eosinophil count <10%. There are differences between the two subtypes of CRSwNP (Cao et al., 2009). Compared with non-ECRSwNP, ECRSwNP is characterized by more eosinophils infiltrating the nasal mucosa, and it has a worse prognosis and higher recurrence rate (Shi et al., 2013). ECRSwNP and non-ECRSwNP have different clinical symptoms, recurrence rates, and responses to drugs and endoscopic surgery (Lou et al., 2015 ). ECRSwNP is a hard-to-treat subtype of CRS.

To discover the pathogenesis of and better treatment for CRSwNP, more research is needed to further explore the different molecular and cytological mechanisms of the subtypes that lead to the different clinical and pathophysiological characteristics between the two subtypes.

With advancements in genomic microarray technology, a revolutionary change has taken place in the field of genetic analysis, that makes it possible to quantify thousands of gene expressions simultaneously. Studies have shown that the human genome can be widely transcribed into a large amount of non-coding RNAs (ncRNAs) that are closely related to the initiation as well as progression of diseases (Beermann et al., 2016). Genomic microarray technology has been widely used in the field of biomedicine to explore the occurrence and development of human diseases, including CRSwNP, at the genetic level (Plager et al., 2010; Yao et al., 2019).

CircRNAs (circular RNAs) are a type of ncRNA with important functions that have tissue specificity and disease specificity (Xia et al., 2017). Unlike linear RNAs (containing 5′ and 3′ ends), circRNAs are closed continuous loops that are free from exonuclease-mediated degradation and are more stable than most linear RNAs (Jeck et al., 2013). It has been found that circRNAs, acting as miRNA sponges, are rich in miRNA binding sites and increase the expression of target genes by mitigating the inhibition of miRNAs on their target genes (Kulcheski et al., 2016). CircRNAs represent a class of naturally occurring endogenous ncRNAs that have recently been recognized as important regulators of gene expression networks (Oude Voshaar et al., 2019). In recent years, researchers have explored the expression profiles of circRNAs in different diseases. For example, one study showed that oxidized low-density lipoprotein accelerates the injury of endothelial cells via the circ-USP36/miR-98-5p/VCAM1 axis (Peng et al., 2021). Another study found that circRNA_09505 aggravates inflammation and joint damage in RA via the miR-6089/AKT1/NF-κB axis (Yang et al., 2020).

MiRNAs (microRNAs) are another group of ncRNAs that are involved in many pathologic and physiological processes, such as proliferation, differentiation, and tumorigenesis (Zhang X.-H. et al., 2012; Ferreira et al., 2018; Martínez-Rivera et al., 2018). Research has shown that the expression of miR-125 b is increased in ECRSwNP, which may lead to mucosal eosinophilia (Zhang Y.-N. et al., 2012). In addition, miR-1 can regulate the transport of eosinophils in CRS. Overexpression of miR-1 inhibits the increase of airway eosinophils and inhibits the binding of eosinophils and endothelial cells induced by IL-13 (Korde et al., 2020). The interaction between circRNAs and miRNAs plays an important role in inflammation and immune responses. In psoriasis, circRNA-0061012 enhances GAB1 expression through spongy miR-194-5p, thereby promoting the proliferation, migration, and invasion of keratinocytes induced by IL-22 (He et al., 2021). CircRNA-WBSCR17 aggravates the inflammatory response of human renal tubular epithelial cells induced by high glucose by targeting the miR-185-5p/SOX6 axis (Li et al., 2020). In osteoarthritis, circRNA-9119 blocks the miR-26a/PTEN axis to protect IL-1β-treated chondrocytes from apoptosis (Chen et al., 2020). These reports show that circRNAs and miRNAs are related to inflammation.

To date, dysregulated expression of miRNAs has been shown in psoriasis, rheumatoid arthritis, pulmonary fibrosis, and allergic asthma. CircRNA is also involved in inflammatory diseases, but there is no research of circRNA in CRSwNP (Zhang Y.-N. et al., 2012). In our study, we aimed to compare the microarray expression profiles of miRNAs and circRNAs in nasal polyps of CRSwNP and normal nasal mucosa from control subjects. However, the results of RNA-seq genomic microarray analysis of ECRSwNP and non-ECRSwNP had no statistical differences, so, we combined ECRSwNP and non-ECRSwNP into a single group denoted as CRSwNP. Then, we validated the abnormally expressed circRNAs and miRNAs by qRT-PCR (quantitative real time polymerase chain reaction). In particular, we explored the potentially biological functions and involved signaling pathways of these ncRNAs by using the GO and KEGG databases. We concluded that the differentially expressed circRNAs and miRNAs may play important roles in the pathogenesis of CRSwNP. Based on ROC (receiver operating characteristic) curve analysis and principal component analysis (PCA), the altered expressions of hsa-circ-0031593, and hsa-miR-145-5p have the most evidence supporting their involvement in the occurrence and development of CRSwNP.

## Materials and Methods

### Subjects and Samples

Nasal polyp specimens were collected from CRSwNP patients undergoing functional endoscopic sinus surgery. The middle turbinate mucosae of the control group were obtained from patients undergoing optic nerve decompression and nasal bone fracture surgery. Controls with nasal inflammation or upper respiratory tract infection were excluded. Subjects using corticosteroids or other immune-modulating drugs within 1 month, and subjects with antrochoanal polyps or fungal sinusitis were excluded. All of the participants were enrolled from the Department of Otorhinolaryngology Head and Neck Surgery, The First Affiliated Hospital of Nanchang University, Nanchang, China in 2017 (detailed information is shown in Supplementary Table S1). The study was approved by the Medical Research Ethics Committee of The First Affiliated Hospital of Nanchang University (2017080).

### Study Process

The quantity of eosinophils in each specimen was observed by hematoxylin-eosin staining in three random microscopic high power fields (HPFs, ×400 magnification). CRSwNP patients were classified according to the percentage of eosinophils in nasal polys. Nasal polyps from three ECRSwNP, three non-ECRSwNP, and three control individuals were collected for total RNA extraction and microarray analysis. We found that ECRSwNP and non-ECRSwNP tissues shared similar expression patterns of circRNAs and miRNAs. Therefore, we combined ECRSwNP and non-ECRSwNP into a single group denoted as CRSwNP. Then, the aberrant circRNAs and miRNAs with fold change > ± 2.5 and *p* < 0.05 were validated in an independent cohort (control group, *n* = 5; CRSwNP, *n* = 5) by qRT-PCR. Next, we expanded the sample size (control group, *n* = 25; CRSwNP group, *n* = 29) to conduct further research on the quantitative expression of the selected ncRNAs in the third cohort.

### Hematoxylin-Eosin Staining for Eosinophils

The quantity of eosinophils was analyzed by hematoxylin-eosin staining. Specimens of nasal polyps were fixed in 10% formaldehyde and placed in low to high concentration alcohol to remove water from the tissues. Then the specimens were embedded in paraffin wax, sliced by a microtome into sections no more than 0.5 μm thick, and deparaffinized to yield tissue sections. After rehydration, the tissue sections were stained with hematoxylin for 10 min and eosin for 3 min. In order to classify CRSwNP into ECRSwNP and non-ECRSwNP, we calculated the percentage of eosinophils in all of the inflammatory cells through five random high-power fields. Specimens with eosinophils ≥10% were defined as ECRSwNP, and those with eosinophils <10% as non-ECRSwNP (AI-Sayed et al., 2017).

### CircRNA Microarray Analysis

The total RNAs were extracted from the subjects for microarray analysis. The purity and concentration of RNA were determined by the OD260/280 readings of a spectrophotometer (NanoDrop ND-1000). The integrity of RNAs was detected by standard denaturing agarose gel electrophoresis (Bioanalyzer 2100, Agilent Technologies, United States). The results are shown in Supplementary Figure S1. The digestion, amplification, and labeling of RNAs were performed based on the protocol provided by the manufacturer. The labeled RNAs were hybridized onto the microarray (Agilent-084217) after purification. The circRNA array data were analyzed by GeneSpring software V13.0 (Agilent). In order to select the differential expression of circRNAs, we used the threshold ≥ ±2.5 fold change and *p* < 0.05.

### MiRNA Microarray Analysis

MiRNA expression profile microarrays of these specimens were performed by CapitalBio. Procedures are described in detail on the CapitalBio website (http://www.capitalbio.com). Briefly, the procedure included total RNA extraction, quality control, miRNA isolation, FlashTag biotin labeling of miRNAs, hybridization to an Affymetrix GeneChip microarray (Affymetrix miRNA 4.0), and microarray washing, staining, and scanning. If the miRNAs expression changed by at least ±2.5-fold (*p* < 0.05), this was considered a significant difference.

### Correlation and Co-Expression Analysis

CircRNAs bind with miRNAs competitively, which inhibits the negative regulation of miRNAs on target genes and leads to the increase of the functional activity and expression of target genes (Fokkens et al., 2012). We constructed co-expression networks to predict the target genes of circRNAs and miRNAs of CRSwNP. The co-expression analysis was based on miRanda-3.3 software, with entropy values below 20. The top 40 circRNA-miRNA networks, *p* < 0.05, were selected for analysis. In the network analysis, each point represents a gene, and two points connected by a line represent two closely related genes.

### Gene Ontology and Kyoto Encyclopedia of Genes and Genomes Pathway Analyses

We identified functional categories that were significantly enriched relative to the background reference by GO enrichment analysis. The related pathways and gene interactions associated with the abnormal expression of circRNA and microRNAs were found based on the latest KEGG pathway enrichment database. The significant GO terms and pathways were determined by Fisher’s exact test, and the false discovery rate was utilized to correct the *p*-values.

### Quantitative Real-Time Polymerase Chain Reaction for Validation of circRNAs and miRNAs

The significant differential expression of circRNAs and miRNAs was quantified by qRT-PCR. Total RNA was isolated from nasal polyps with TRIzol reagent. The cDNAs were synthesized by reverse transcription with a PrimeScript RT reagent kit with random primers. Then, qRT-PCR was conducted by SYBR Premix Ex Taq II (Tli RNaseH Plus; TaKaRa). Primers for selected ncRNAs and house-keeping genes were synthesized by Sangon Biotech (Shanghai, China). The primers used are shown in Supplementary Tables S2, S3.

### Statistical Analysis

SPSS 22.0 was used in this study. The Mann-Whitney U-test was used to calculate the differences of the expression of circRNAs and miRNAs between groups, and *p* < 0.05 was considered to be statistically significant. The functional values of the selected circRNAs and miRNAs for CRSwNP were evaluated by conducting ROC curve analysis and PCA.

## Results

### ECRSwNP and Non-ECRSwNP Patients Shared Similar Gene Expression Profiles

**FIGURE 1:**
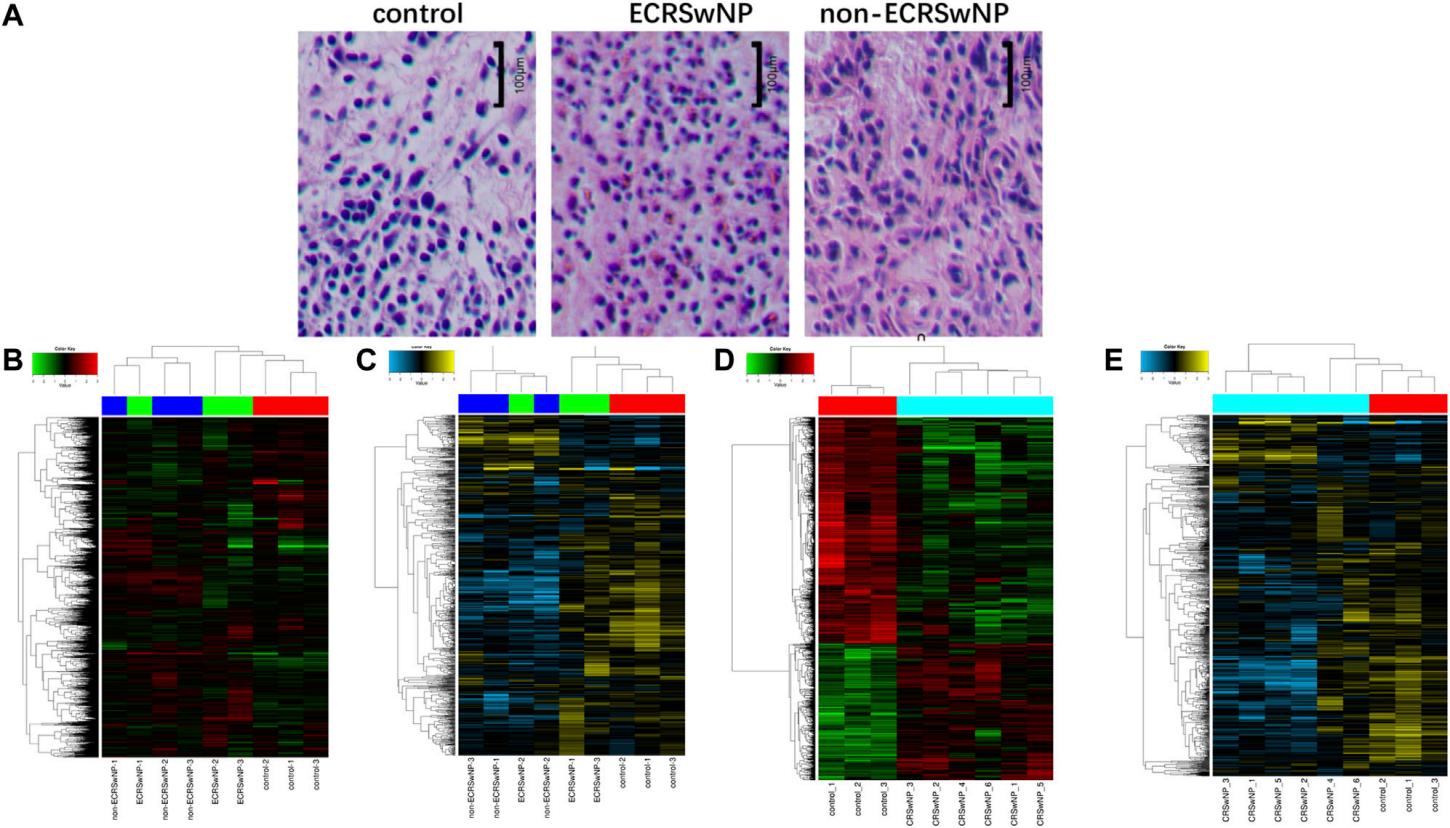
Hematoxylin-eosin staining of control, ECRSwNP, and non-ECRSwNP groups, and differential expression of circRNAs and miRNAs in the three groups. **(A)** Representative images of hematoxylin-eosin staining. The scale bar represents 100 μm. **(B)** Hierarchical clustering analysis heat map showing significantly changed of circRNAs and **(C)** of miRNAs with fold change ≥2.0 (*p* < 0.05) in the three groups (ECRSwNP, non-ECRSwNP, and control). The expressions of circRNAs and miRNAs were significantly different between CRSwNP and control groups, but ECRSwNP and non-ECRSwNP groups shared similar gene expression profiles. Then, they were combined as the CRSwNP group. Hierarchical clustering analysis heat map showing significantly changed of circRNAs **(D)** and of miRNAs **(E)** with fold change ≥2.0 (*p* < 0.05) in the two groups (CRSwNP and control).

In this study, we used hematoxylin-eosin staining for eosinophil counts (Figure 1A). Hierarchical clustering analysis was used for evaluating gene expression differences among groups. As shown in Figures 1B,C, the ECRSwNP and non-ECRSwNP groups shared similar gene expression profiles, so we could not distinguish ECRSwNP from non-ECRSwNP by hierarchical clustering analysis. Then, we combined the ECRSwNP group and non-ECRSwNP group into the CRSwNP group. The results showed that the expression profiles of circRNAs (Figure 1D) and miRNAs (Figure 1E) were significantly different between the CRSwNP and control groups.

### Differential Expression of circRNAs and miRNAs in CRSwNP

**FIGURE 2:**
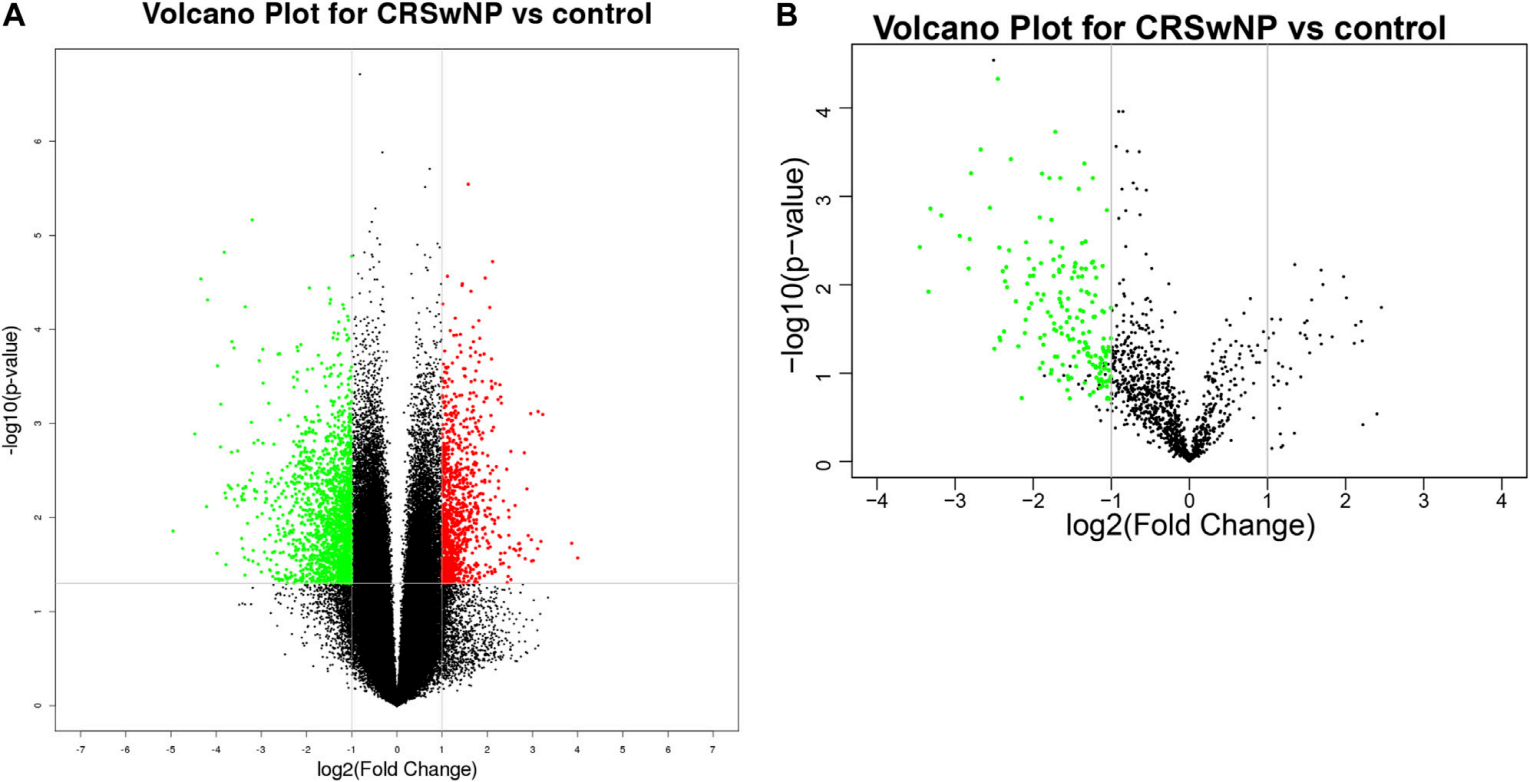
Different expressions of circRNAs and miRNAs shown in volcano plots. The circRNAs and miRNAs were widely distributed in all chromosomes. Each column corresponds to a circRNA or a miRNA, the green column toward the center represents significantly downregulated and the red one toward the outside means upregulated. **(A)** Differential expression of circRNAs. **(B)** Differential expression of miRNAs.

Volcano plots were used to assess the locations of circRNAs (Figure 2A) and miRNAs (Figure 2B). These ncRNAs were widely distributed in all of the chromosomes. Circos plots and scatter plots were also applied to analyze the gene expression differences between the CRSwNP and control groups (Supplementary Figures S2, S3). CircRNAs and miRNAs downregulated or upregulated with fold change ≥ ± 2.5 (*p* < 0.05) in both the ECRSwNP group and non-ECRSwNP group were considered to have significant differential expression and were selected for further research. A total of 2,875 circRNAs showed significant differential expression in the CRSwNP group, including 1,794 downregulated circRNAs and 1,081 upregulated circRNAs. Additionally, 192 miRNAs were significantly downregulated and no miRNAs were significantly upregulated in the CRSwNP group.

### Co-Expression Network in CRSwNP

**FIGURE 3:**
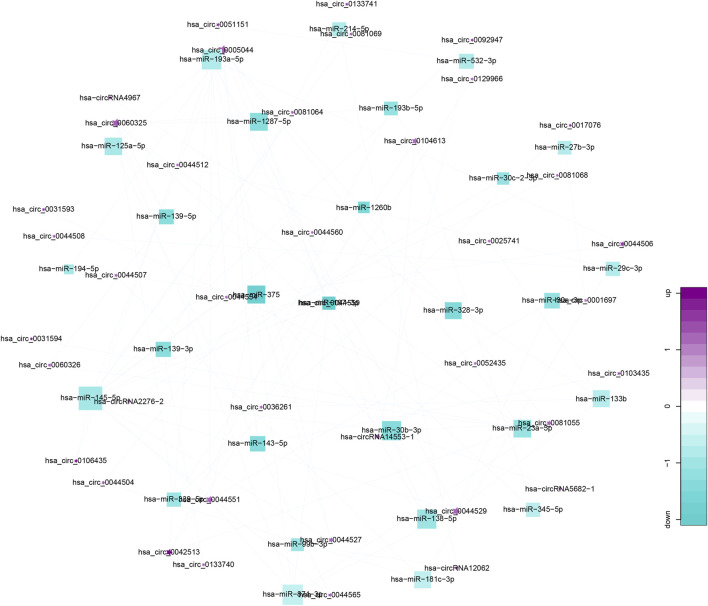
Top 40 circRNA-miRNA co-expression networks. Co-expression networks were analyzed by miRanda-3.3 software, combined with entropy values below 20. Stars represent circRNAs; squares represent miRNA; lines represent correlative relationships. Different sizes and colors represent the corresponding (up/down) relationship.

From gene co-expression network analysis, we found that there was a difference in the co-expression of circRNAs and miRNAs between the CRSwNP group and control group, which revealed the underlying molecular mechanism of the pathogenesis of CRSwNP. We selected 40 circRNAs differentially expressed between the CRSwNP and control groups. The top 40 circRNAs-miRNAs networks are shown in Figure 3. Hsa-circ-0031593, hsa-circ-0031594, and hsa-miR-27b-3p are present in Figure 3. One circRNA can be associated with multiple miRNAs, and one miRNA can be related to multiple circRNAs, resulting in complex functional connections. In Figure 3, the darker and larger nodes indicate the higher fold change of circRNAs, purple indicates upregulation, and blue indicates downregulation. There were many regulatory relationships between the circRNAs and miRNAs in the networks, which further indicated that the networks of regulatory relationships were ubiquitous.

### Gene Ontology and Kyoto Encyclopedia of Genes and Genomes Pathway Analyses

**FIGURE 4:**
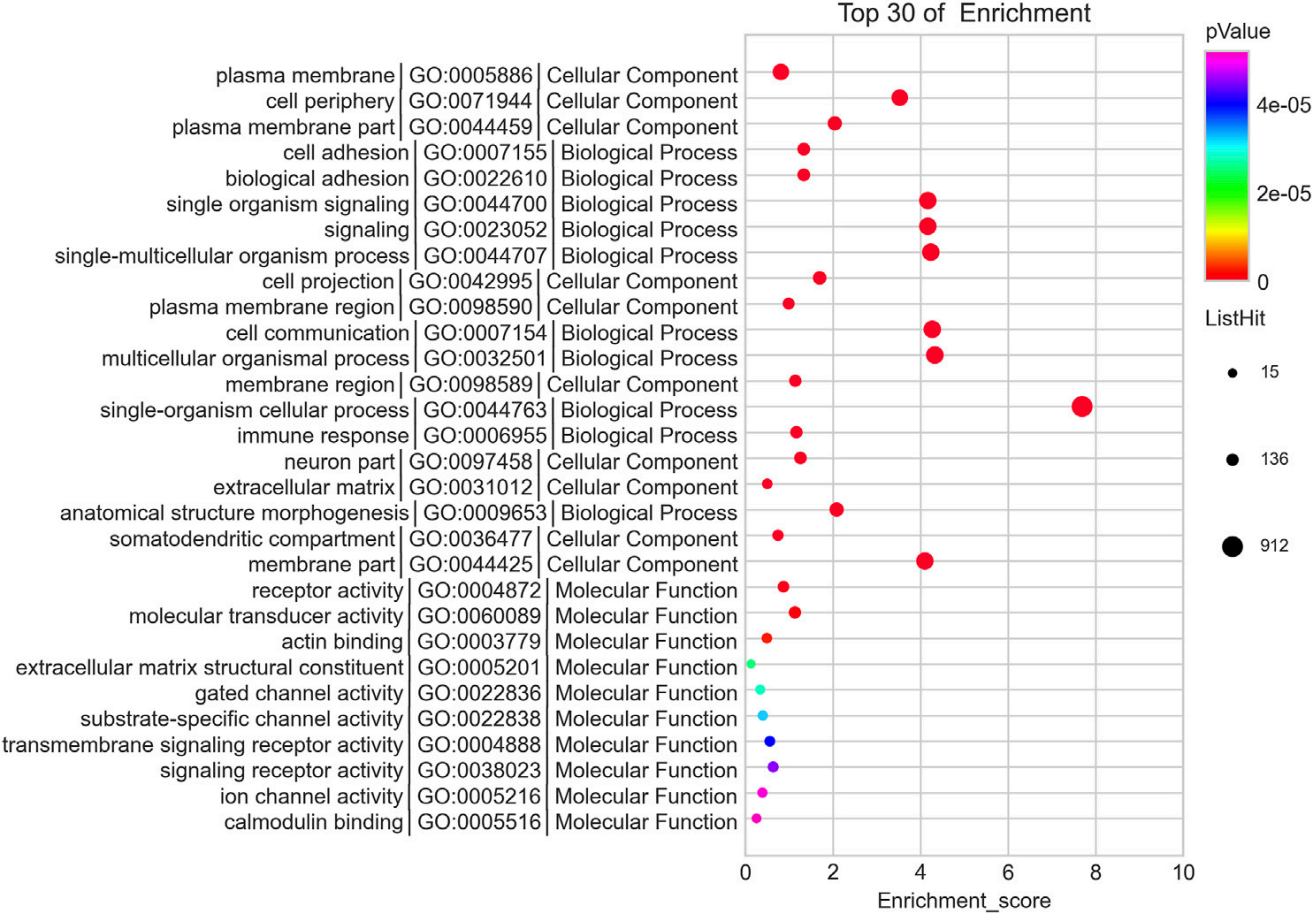
Top 10 significant enriched GO terms analysis of circRNAs. Differentially expressed circRNAs were mainly involved in biological process, cellular component, and molecular function. Each plot represents a specific biological function, and the larger the size, the stronger the circRNAs expression. Compared with the normal subjects, the significantly over-presented circRNAs in the CRSwNP group were mainly involved in cell adhesion, membrane, and receptor activity.

**FIGURE 5:**
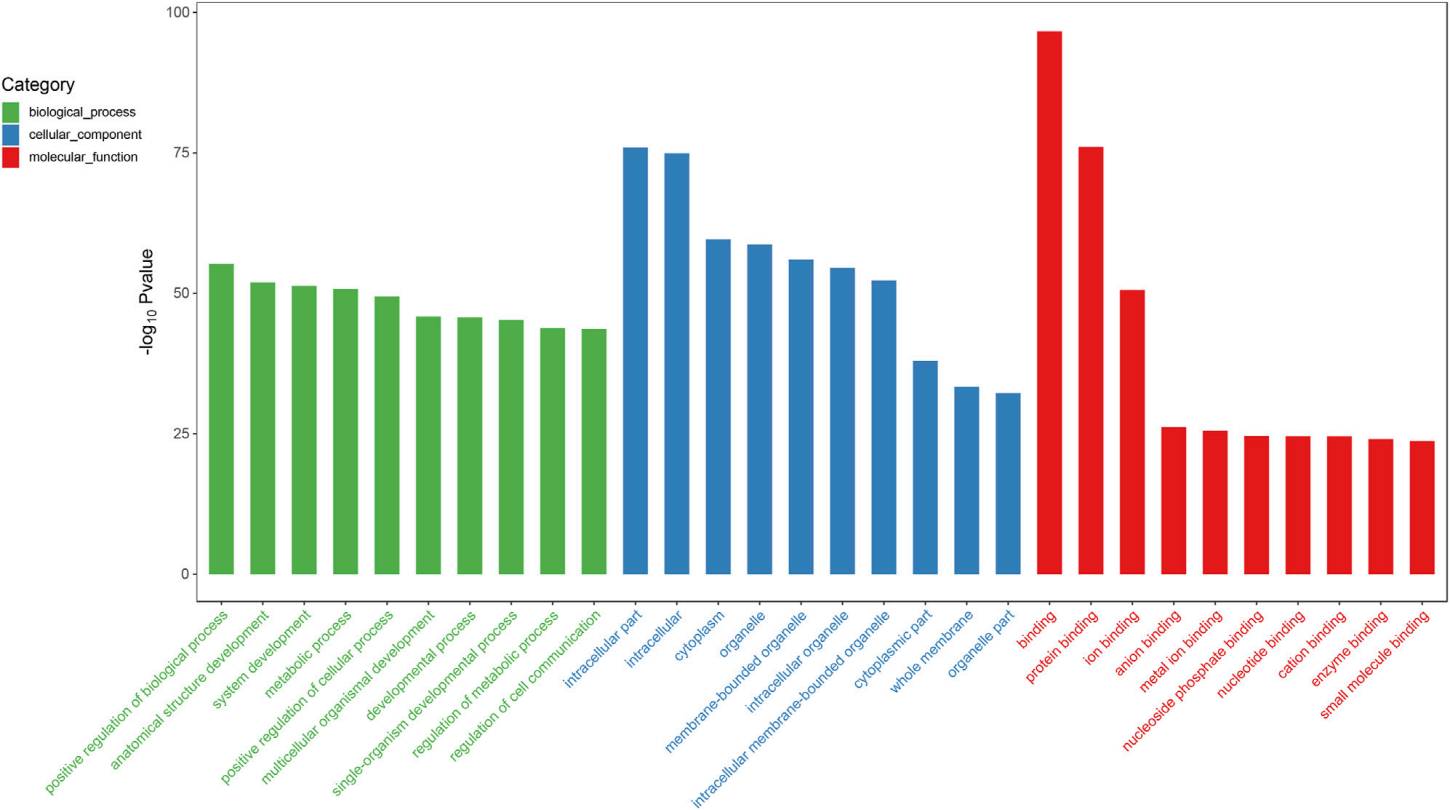
Top 10 significant enriched GO terms analysis of miRNAs. Differentially expressed miRNAs were mainly involved in biological process, cellular component, and molecular function. Compared with the normal subjects, the significantly over-presented miRNAs in the CRSwNP group were mainly involved in positive regulation of biological process, intracellular part, and binding.

**FIGURE 6:**
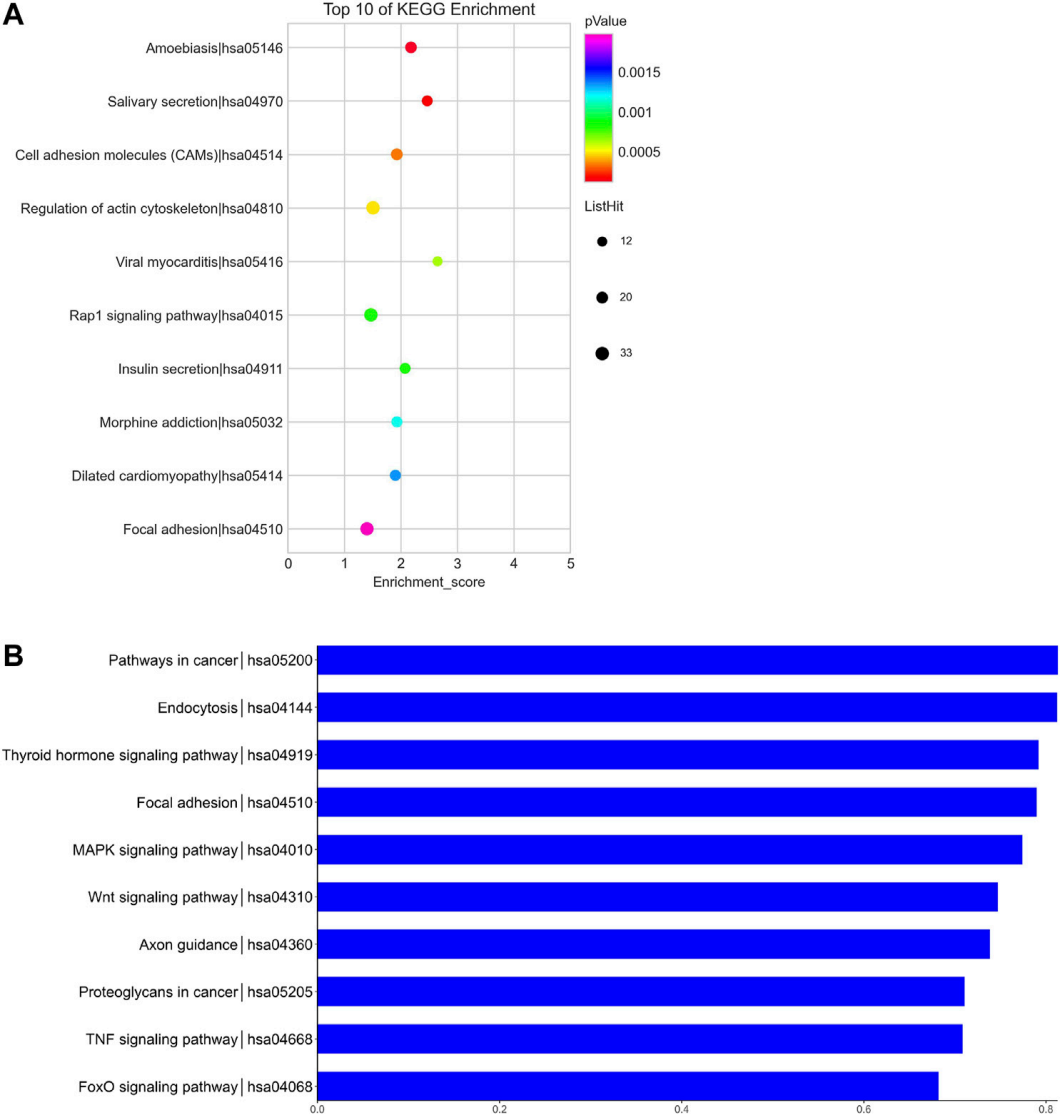
KEGG pathway analysis of circRNAs and miRNAs. Biological roles of the differentially expressed circRNAs **(A)** and miRNAs **(B)**. Biological roles of the differentially expressed circRNAs include amebiasis, salivary secretion, cell adhesion molecules (CAMs), and so on. Biological roles of the differentially expressed miRNAs include pathways in cancer, endocytosis, thyroid hormone signaling pathway, and so on.

We predicted the biological functions of aberrantly expressed circRNAs and miRNAs through GO enrichment analysis and KEGG pathway analysis. The enriched GO terms (*p* < 0.05) could reflect the function of aberrantly expressed circRNAs and miRNAs. Each plot represents a specific biological function, and a larger size means a larger gene number. For circRNAs, compared with the normal control subjects, the significantly over-presented terms were mainly involved in cell adhesion, membrane, and receptor activity. In contrast, miRNAs were mainly involved in cellular component organization, intracellular parts, protein binding, and so on. The details of the top 10 significantly enriched GO terms of circRNAs are presented in Figure 4. According to GO analysis of circRNAs in the biological process (BP) category, the differentially expressed circRNAs were chiefly enriched in “single-organism process” and “single-organism cell process” (Figure 4). GO analysis related to cellular components (CC) showed that they were mainly enriched in “membrane” and “membrane part” (Figure 4). GO analysis related to molecular functions (MF) demonstrated that they were involved in “molecular transducer activity” and “receptor activity” (Figure 4). The GO analysis of miRNAs is shown in Figure 5. The biological process category was enriched in “positive regulation of biological process” and “anatomical structure development” (Figure 5). Cellular components (CC) were mostly enriched in “intracellular” and “intracellular part” (Figure 5). Molecular functions (MF) were involved in “binding” and “protein binding” (Figure 5). In addition, from KEGG pathway analysis, we found that differentially expressed circRNAs were annotated to amebiasis, salivary secretion, and others (Figure 6A); and miRNAs were annotated to pathways in cancer, endocytosis, and so on (Figure 6B).

### Confirmation of the Expression of circRNAs and miRNAs Using RNA-Sequencing and Quantitative Real-Time Polymerase Chain Reaction

**FIGURE 7:**
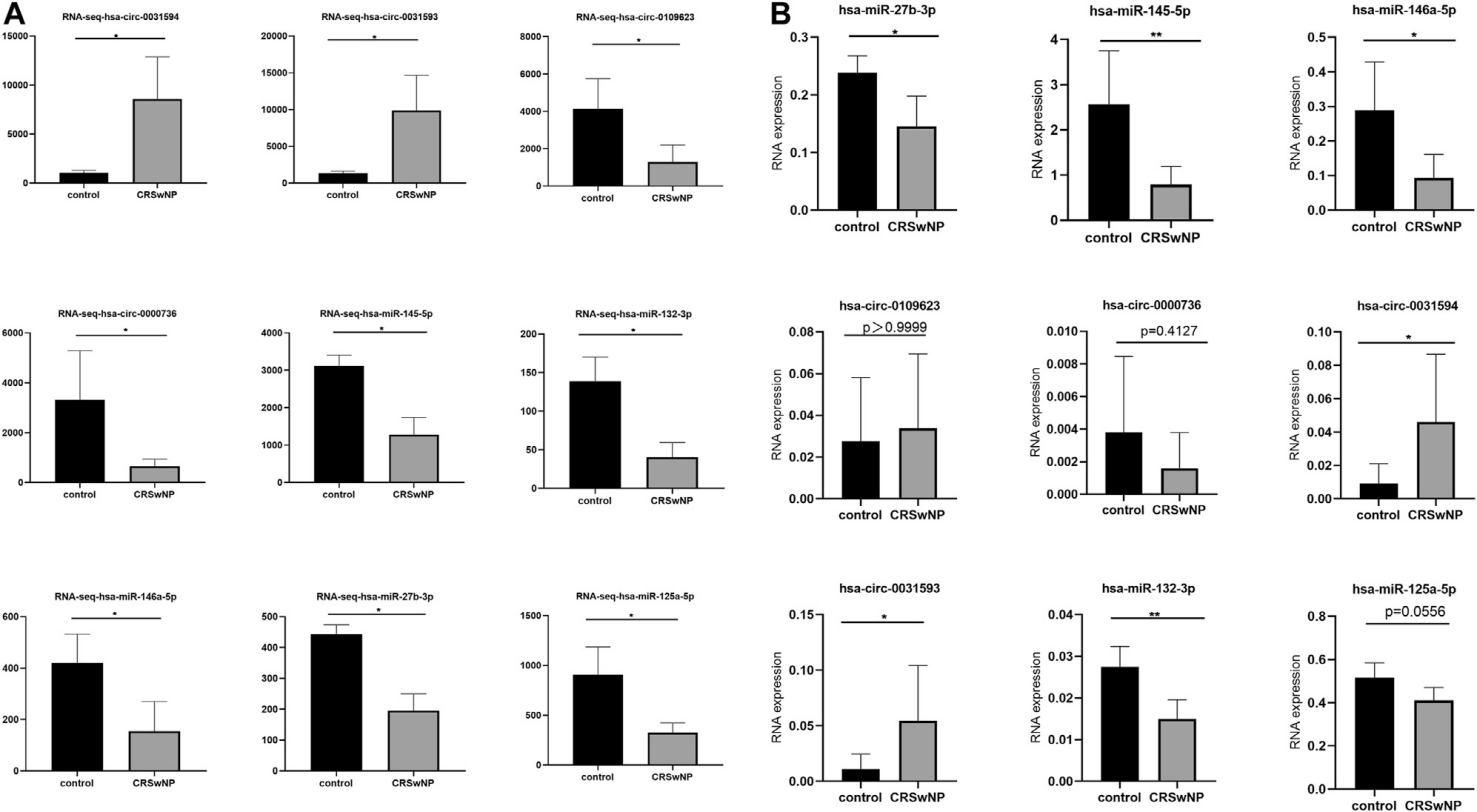
Confirmation of the expression of circRNAs and miRNAs using RNA-sequencing and quantitative real-time polymerase chain reaction (qRT-PCR). CircRNAs and miRNAs were validated by RNA-sequencing (CRSwNP(*n* = 6); control subjects (*n* = 3) **(A)**; and qRT-PCR in an independent cohort of five CRSwNP subjects and five control subjects **(B)**. Each sample was detected in triplicate. GAPDH was used as the reference gene for circRNAs, and hsa-miR-16 was used as the reference gene for miRNAs. The relative expression levels of hsa-circ-0031593, hsa-circ-0031594, hsa-circ-0109623, hsa-circ-0000736, hsa-miR-132-3p, hsa-miR-145-5p, hsa-miR-146a-5p, hsa-miR-125a-5p, and hsa-miR-27b-3p by qRT-PCR. *p < 0.05, **p < 0.01, ***p < 0.001, ****p < 0.0001.

The results of RNA-seq analysis showed that hsa-circ-0031593 and hsa-circ-0031594 were expressed to a significantly higher degree in the CRSwNP group than in the control group. Hsa-circ-0109623, hsa-circ-0000736, hsa-miR-125a-5p, hsa-miR-132-3p, hsa-miR-145-5p, hsa-miR-146a-5p, and hsa-miR-27b-3p were to a significantly lower degree in the CRSwNP group than in the control group (Figure 7A). To confirm the reliability of the microarray results, circRNAs and miRNAs downregulated and upregulated with fold change ≥ ± 2.5 (*p* < 0.05) in five CRSwNP subjects and five control subjects were selected for qRT-PCR in order to analyze the expression levels of these ncRNAs. The results showed that hsa-circ-0031593 and hsa-circ-0031594 were expressed significantly more in the CRSwNP group than in the control group. Hsa-miR-132-3p, hsa-miR-145-5p, hsa-miR-146a-5p, and hsa-miR-27b-3p were expressed significantly less in the CRSwNP group than in the control group. There were no statistical differences among the expressions of hsa-circ-0109623, hsa-circ-0000736, or hsa-miR-125a-5p (Figure 7B ).

**FIGURE 8:**
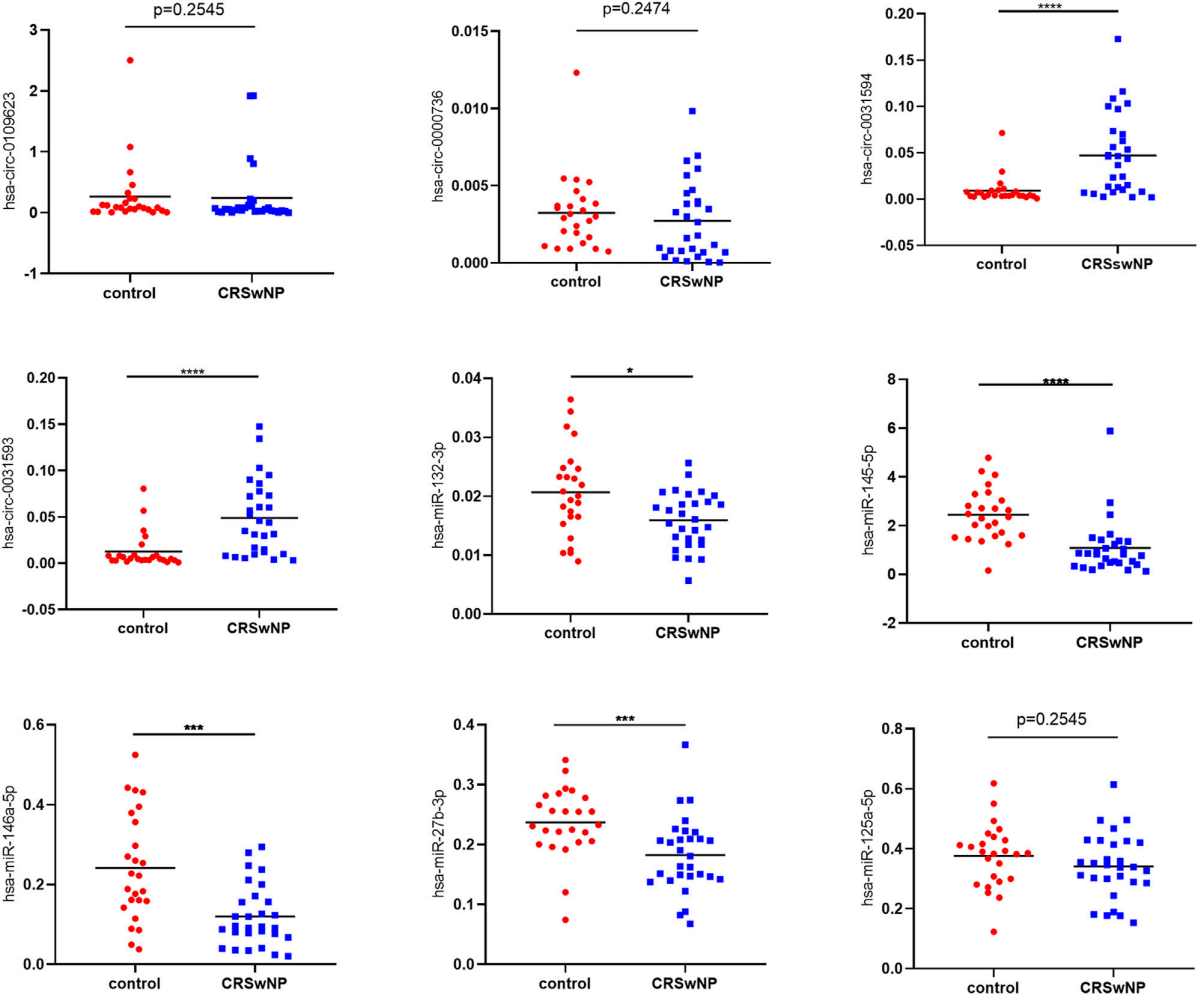
Confirmation of the expression of circRNAs and miRNAs using quantitative real-time polymerase chain reaction (qRT-PCR). Sample numbers were expanded to perform qRT-PCR (CRSwNP (*n* = 29); control subjects (*n* = 25). GAPDH was used as the reference gene for circRNAs, and hsa-miR-16 was used as the reference gene for miRNAs. The heights of columns represent the fold changes of CRSwNP compared with the control group. The relative expression levels of hsa-circ-0031593, hsa-circ-0031594, hsa-circ-0109623, hsa-circ-0000736, hsa-miR-132-3p, hsa-miR-145-5p, hsa-miR-146a-5p, hsa-miR-125a-5p and hsa-miR-27b-3p by qRT-PCR. *p < 0.05, **p < 0.01, ***p < 0.001, ****p < 0.0001.

After that, more samples (29 CRSwNP subjects and 25 control subjects) were used for further research of the quantitative expressions of these circRNAs and miRNAs. The detailed expression levels of these ncRNAs are shown in Figure 8. The results in Figure 8 are in accordance with the results in Figure 7B . The expressions of hsa-circ-0031593 and hsa-circ-0031594 in the CRSwNP group were significantly higher than those in the control group. The expressions of hsa-miR-132-3p, hsa-miR-145-5p, hsa-miR-146a-5p, and hsa-miR-27b-3p in the CRSwNP group were significantly lower than those in the control group. There were no statistical differences among the expressions of hsa-circ-0109623, hsa-circ-0000736, or hsa-miR-125a-5p.

### ROC Curve Analysis and PCA of Selected circRNAs and miRNAs

**FIGURE 9:**
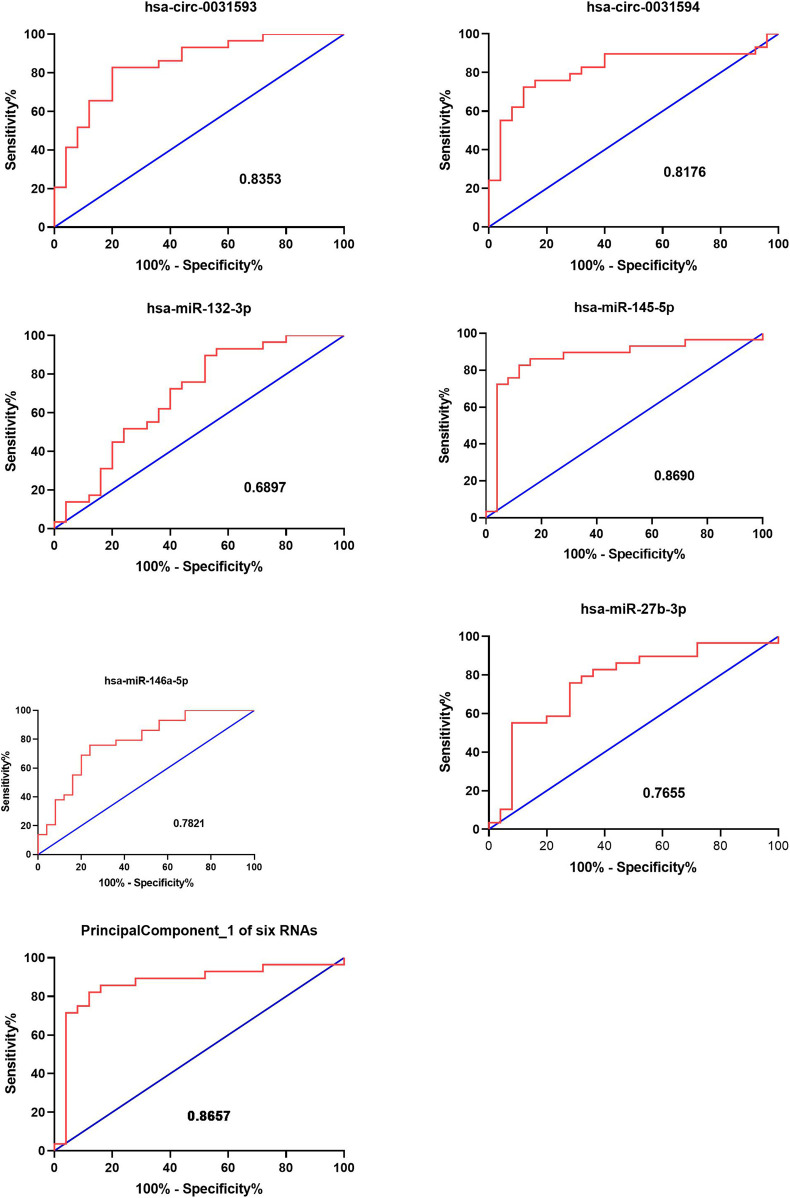
Functional value of circRNAs and miRNAs. The receiver operating characteristic (ROC) curve analysis for the function of CRSwNP. The ROC curve analysis of hsa-circ-0031593, hsa-circ-0031594, hsa-miR-132-3p, hsa-miR-145-5p, hsa-miR-146a-5p, and hsa-miR-27b-3p and principal component 1 of these six ncRNAs for the significance in the occurrence and development of CRSwNP. The AUC (area under curves) values are given on the graphs.

ROC curve analysis was carried out to estimate the functional values of the selected circRNAs and miRNAs in the occurrence and development of CRSwNP (Figure 9). The sensitivity and specificity for the values of CRSwNP are shown in Table 1. The PCA method was used in this research. The data of PCA with combinations of hsa-circ-0031593, hsa-circ-0031594, hsa-miR-132-3p, hsa-miR-145-5p, hsa-miR-146a-5p, and hsa-miR-27b-3p are displayed in Table 2. The first principal component explained variance ratio was 98.87%. We used the first principal component 1 of these six ncRNAs to carry out ROC curve analysis, and the AUC was 0.8657, indicating a good significance for the pathogenesis of CRSwNP. The AUCs of hsa-circ-0031593 and hsa-miR-145-5p were 0.8353 [(0.7291–0.9415), *p* < 0.0001] and 0.8690 [(0.76–0.978), *p* < 0.0001]. Hsa-circ-0031593 and hsa-miR-145-5p had the strongest evidence supporting their involvement in the occurrence and development of CRSwNP since they had higher AUCs than others and had *p* values < 0.05.

**TABLE 1:** Validation of the selected circRNAs and miRNAS by quantitative real-time polymerase chain reaction and the data of ROC curve analysis.

	AUC	95% CI	*p* value	Sensitivity	Specificity
Hsa-circ-0031593	0.8353	0.7291–0.9415	<0.0001	0.8235	0.80
Hsa-circ-0031594	0.8176	0.7047–0.9306	<0.0001	0.7059	0.88
Hsa-miR-132-3p	0.6897	0.5438–0.8355	0.0171	0.8966	0.48
Hsa-miR-145-5p	0.8690	0.76–0.978	<0.0001	0.8276	0.88
Hsa-miR-146a-5p	0.7821	0.6579–0.9063	0.0004	0.7586	0.76
Hsa-miR-27b-3p	0.7655	0.6332–0.8979	0.0008	0.7586	0.72
Principal component 1 of six RNAs	0.8657	0.7547–0.9768	<0.0001	0.8214	0.88

AUC, area under curves.

**TABLE 2:** The data of principal component 1 by PCA with combinations of hsa-circ-0031593, hsa-circ-0031594, hsa-miR-132-3p, hsa-miR-145-5p, hsa-miR-146a-5p, and hsa-miR-27b-3p.

Principal component 1	Type of sample
−0.207152043	Control
2.500343003	Control
0.987185596	Control
1.304084045	Control
−0.361852501	Control
0.755023033	Control
−1.570053914	Control
−0.481308455	Control
0.906674823	Control
0.30217685	Control
2.353877347	Control
1.965290304	Control
−0.28100719	Control
1.564228751	Control
3.054599405	Control
−0.126055013	Control
0.567948512	Control
1.087283413	Control
−0.009538338	Control
0.62720042	Control
0.255341755	Control
−0.162273621	Control
0.38535951	Control
1.632552725	Control
0.966021961	Control
−1.33220639	CRSwNP
−0.361203299	CRSwNP
−0.386665708	CRSwNP
−1.597233545	CRSwNP
−1.396044339	CRSwNP
−1.205543467	CRSwNP
−1.376653623	CRSwNP
4.157249255	CRSwNP
−0.902849991	CRSwNP
−0.860962834	CRSwNP
−0.22755494	CRSwNP
−1.464884451	CRSwNP
−0.876447159	CRSwNP
0.712907312	CRSwNP
−0.962998826	CRSwNP
1.21901289	CRSwNP
−0.685225616	CRSwNP
−1.19975492	CRSwNP
−1.560321	CRSwNP
−0.314747233	CRSwNP
−0.088432428	CRSwNP
−1.263311269	CRSwNP
−0.777328458	CRSwNP
−1.238227185	CRSwNP
−0.490926392	CRSwNP
−1.537602896	CRSwNP
−0.910574069	CRSwNP
−1.087419795	CRSwNP

## Discussion

CRSwNP is a significant public health problem with a considerable socioeconomic burden. Previous studies have reported that CRSwNP is a complex, multifactorial disease. There have been many studies on its etiology, but its pathogenesis remains unclear. Dysregulated expression of miRNAs has been shown in psoriasis, rheumatoid arthritis, pulmonary fibrosis, and allergic asthma. CircRNA is also involved in inflammatory diseases, but there has been no research on the role of circRNA in CRSwNP. Although numerous researchers have attempted to clarify the pathogenesis of CRSwNP, the detailed mechanisms remain unclear. Overall, little is known on the role of ncRNAs in the pathogenesis of CRSwNP. Further understanding of the genetic level of pathogenesis is essential for developing new techniques for effective prevention and therapy to improve prognosis. Researchers have found that ncRNAs, such as circRNAs and miRNAs, play essential roles in the occurrence and development of many diseases, which is contrary to the traditional view that genes are mainly regulated by protein coding (Wu et al., 2019; Yang et al., 2019). To explore the functions of circRNAs and miRNAs in CRSwNP, we performed gene microarray analysis of circRNAs and miRNAs in a CRSwNP group and a control group. Functional enrichment analysis and prediction of differentially expressed genes were carried out by using public databases. In addition, we performed qRT-PCR to validate the reliability of RNA-seq analysis, and we confirmed that hsa-circ-0031593 and hsa-miR-145-5p had the strongest evidence supporting their involvement in the occurrence and development of CRSwNP by ROC curve analysis and PCA. In conclusion, our findings revealed a network potentially involved in CRSwNP pathogenesis, in which circRNAs and microRNAs play significant roles.

ECRSwNP differs greatly from non-ECRSwNP in many aspects (Shah et al., 2016; Lou et al., 2018), such as pathogenesis, development, prognosis, and CT scan images. Our original intention was to explore the functions of miRNA and circRNA in different CRSwNP subtypes, in order to help postoperative treatments like determining the eosinophil count. In addition, some researchers have found that ECRSwNP is difficult to treat and has a high recurrence rate, leading to poor clinical outcomes. However, in our study, the expressions of circRNA and miRNA between ECRSwNP and non-ECRSwNP had no statistical differences. It is possible that individual differences of the samples or the regulation of the next biological process has changed, resulting in different types of polyps. The development process of CRSwNP is complex and diverse (Schleimer, 2017). Meanwhile, the classification between ECRSwNP and non-ECRSwNP is limited. First, there is no unified view on the determination of ECRSwNP throughout the world. Second, the count of eosinophils is objective. Therefore, we combined ECRSwNP and non-ECRSwNP into the CRSwNP group.

After analyzing the different expressions of circRNAs and miRNAs between the CRSwNP and control groups, we found that 1794 circRNAs were significantly downregulated and 1,081 were significantly upregulated in the CRSwNP group. Additionally, 192 miRNAs showed significant downregulation in the CRSwNP group and none showed upregulation. The different expressions of circRNAs and miRNAs between these two groups may be involved in the pathogenesis of CRSwNP.

Studies have shown that one of the important functions of circRNAs is to act as “miRNA sponges” and competitively bind miRNAs to regulate post-transcriptional activity (Ergun and Oztuzcu, 2015). Co-expression networks have been constructed to obtain the relationship between circRNAs and miRNAs. Figure 3 shows that a single circRNA is associated with multiple miRNAs, and a single miRNA is associated with multiple circRNAs. Little is known about the relationship between circRNAs and miRNAs in CRSwNP. In fact, the present study is the first to detect circRNA in CRSwNP.

Without functional analysis, the huge quantity of data on gene expression is unintelligible. Hence, functional enrichment analysis and prediction of differentially expressed genes were carried out using public databases, such as GO enrichment analysis and KEGG pathway analysis, and we found that differential expressions of circRNAs between the CRNwNP and control groups were related to “amoebiasis,” “salivary secretion,” “cell adhesion molecules (CAMs),” “cAMP signaling pathway,” “focal adhesion”, “adherens junction,” “TNF signaling pathway,” and others. The differential expressions of miRNAs between the CRSwNP and control groups were enriched in “pathways in cancer,” “endocytosis,” “thyroid hormone signaling pathway,” “salivary secretion,” “regulation of actin cytoskeleton,” “insulin secretion,” and so on. Based on the functional analysis, the most significantly enriched pathway was CAMs, which was consistent with previous research (Milonski et al., 2015; Xu et al., 2017). The pathological characteristics of CRSwNP include inflammatory cells migrating to and infiltrating the nasal mucosa. During different stages of the progression of CRSwNP, the expressions of CAMs are various, which stimulate eosinophil and mast cell aggregation and contribute to Th2 skewing (Oyer et al., 2013). Compared to normal nasal mucosa, CRSwNP was shown to be more sensitive to IL-32 through lipopolysaccharides acting at the cAMP signaling pathway (Cho et al., 2016). Further, a study found that thromboxane A2 is involved in platelet aggregation and tissue inflammation in CRS, and cAMP regulates the expression of the thromboxane-prostanoid receptor and cxcl1/8, which participates in the pathogenesis of CRS (Elion et al., 2018). TNF, a complex and important inflammatory factor, induces local production of IgA and stimulates eosinophils, and it plays an important role in the pathogenesis of CRSwNP (Kato et al., 2008; Cho et al., 2015; Shimizu et al., 2016). The integrity of the airway epithelium is a prerequisite for its good barrier function, which depends on the intercellular junctions, including tight junctions and adhesion junctions (Suzuki et al., 2016; Jiao et al., 2018). Studies have shown that the breakdown of tight junctions and adhesion junctions of CRS and the decrease of protein component expression are major factors leading to the occurrence of CRS (Kim et al., 2018; Tian et al., 2018). Studies on the key genes and pathways in CRSwNP showed that salivary secretion was the most significantly enriched pathway for downregulated genes, which was consistent with our findings (Yao et al., 2019). Above all, our findings confirmed the validity of previous research and showed high reliability. Little is known about the regulation of the actin cytoskeleton and insulin secretion in CRSwNP. Studies have shown that the actin cytoskeleton is associated with several inflammatory diseases and is involved in leukocyte transendothelial migration (Schnoor, 2015; Ao et al., 2016; Lechuga and Ivanov, 2017). Besides, numerous studies have shown that the actin cytoskeleton plays an important role in regulating insulin secretion (Martínez-García et al., 2015; Sorrenson et al., 2016; Deyev et al., 2017). However, the functions of the actin cytoskeleton and insulin secretion in CRSwNP need to be further explored.

The heterogeneity of CRSwNP is gradually being recognized, prompting the discovery of novel biomarkers to describe specific endotypes and determine optimized treatment (Dennis et al., 2016; Kuhar et al., 2017). Recent studies on potential biomarkers in CRSwNP mainly focused on eosinophils, exhaled gas components, and inflammatory cells in nasal secretions, nasal tissues, and peripheral blood (Drake et al., 2016; Tsybikov et al., 2016; Asano et al., 2017; Chen et al., 2017). Yan indicated that miR-145-5p negatively regulates the proliferation and chemokine secretion of NHEKs by targeting MLK3, and the downregulation of miR-145-5p contributes to skin inflammation in psoriasis lesions (Yan et al., 2019). Dihydroquercetin attenuates lipopolysaccharide-induced acute lung injury by modulating FOXO3-mediated NF-κB signaling via miR-132–3p (Liu J.-H. et al., 2020). Human neutrophil elastase induces MUC5AC overexpression in chronic rhinosinusitis through miR-146a (Yan et al., 2020 ). Furthermore, miR-27b-3p, miR-181a-1-3p, and miR-326-5p are involved in the inhibition of macrophage activation in chronic liver injury (Li et al., 2017). Circ_0134111 knockdown relieves IL-1β-induced apoptosis, inflammation, and extracellular matrix degradation in human chondrocytes through the circ_0134111-miR-515-5p-SOCS1 network (Wu et al., 2021). An inducible circular RNA circKcnt2 inhibits ILC3 activation to facilitate colitis resolution (Liu B. et al., 2020 ). These studies show that circRNA and miRNA play vital functions in the process of inflammation. In the present study, ROC curve analysis and PCA indicated that aberrantly expressed circRNAs and miRNAs may be related to biological dysfunction and play important roles in the pathogenesis of CRSwNP. Furthermore, hsa-circ-0031593 and hsa-miR-145-5p were more likely to correlate with the occurrence and development of CRSwNP.

There are still some limitations in our study. First, individual differences of the samples may have led to the lack of statistical differences between the ECRSwNP and non-ECRSwNP groups, although our findings were consistent with previous research (Cho et al., 2016; Suzuki et al., 2016; Xu et al., 2017; Kim et al., 2018; Yao et al., 2019). Second, all patients’ data were from The First Affiliated Hospital of Nanchang University, and all patient were from Jiangxi Province. Although most of the Chinese population is Han, given that ethnic and regional variations may be involved in the development of CRSwNP, we will consider these variables in future studies. Third, our findings were only based on gene chip analysis, database comparison and prediction, and tissue experiment verification. Experiments *in vivo* and *in vitro* should be carried out to further explore the function of these aberrant genes in CRSwNP.

## Conclusion

In our study, the expression profiles of ECRSwNP and non-ECRSwNP had no statistical differences. The differentially expressed circRNAs and miRNAs between the CRSwNP and control groups may play important roles in the pathogenesis of CRSwNP. Altered expression of hsa-circ-0031593 and hsa-miR-145-5p had the strongest evidence for involvement in the occurrence and development of CRSwNP.

## Data Availability

The datasets presented in this study can be found in online repositories. The name of the repository and accession numbers can be found below: National Center for Biotechnology Information (NCBI) Gene Expression Omnibus (GEO), https://www.ncbi.nlm.nih.gov/geo/, GSE169375 and GSE169376'
