# Supplementary figures and images for: Gene Expression Profiles of Circular RNAs and MicroRNAs in Chronic Rhinosinusitis With Nasal Polyps
Source: Front Mol Biosci. 2021 May 28;8:643504. doi: 10.3389/fmolb.2021.643504 (PMC8194396; doi:10.3389/fmolb.2021.643504)

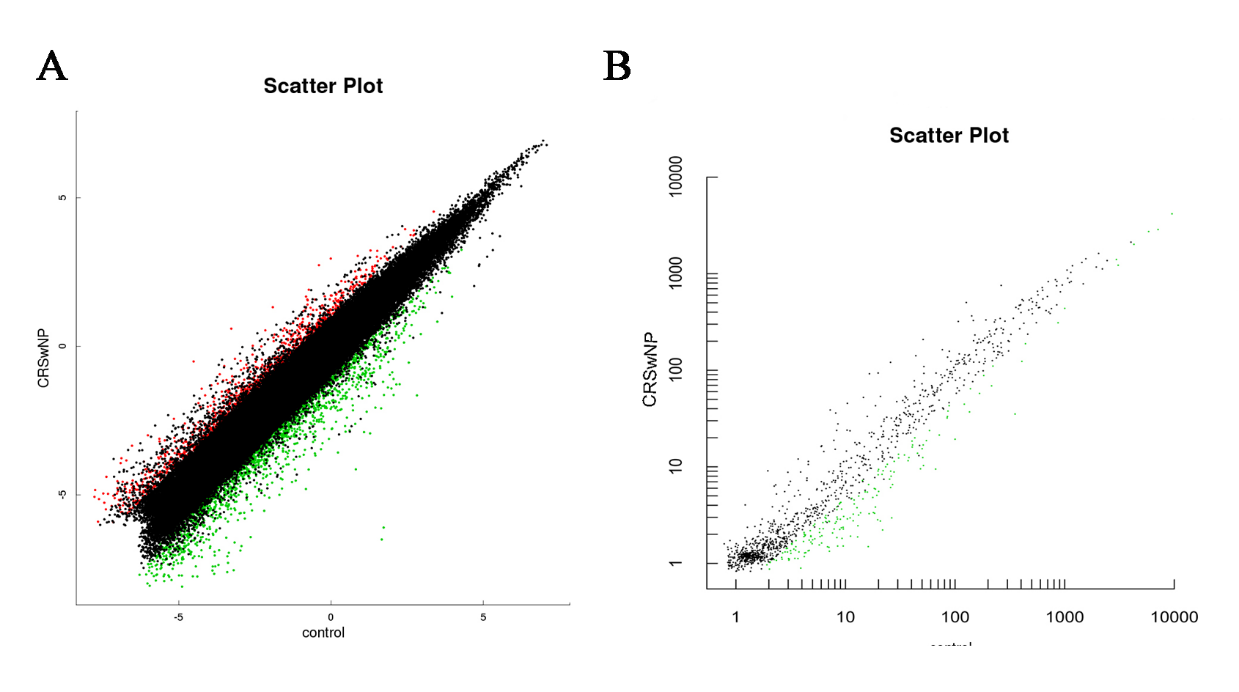

Supplement: Supplementary file 1 [file Image3.TIF]

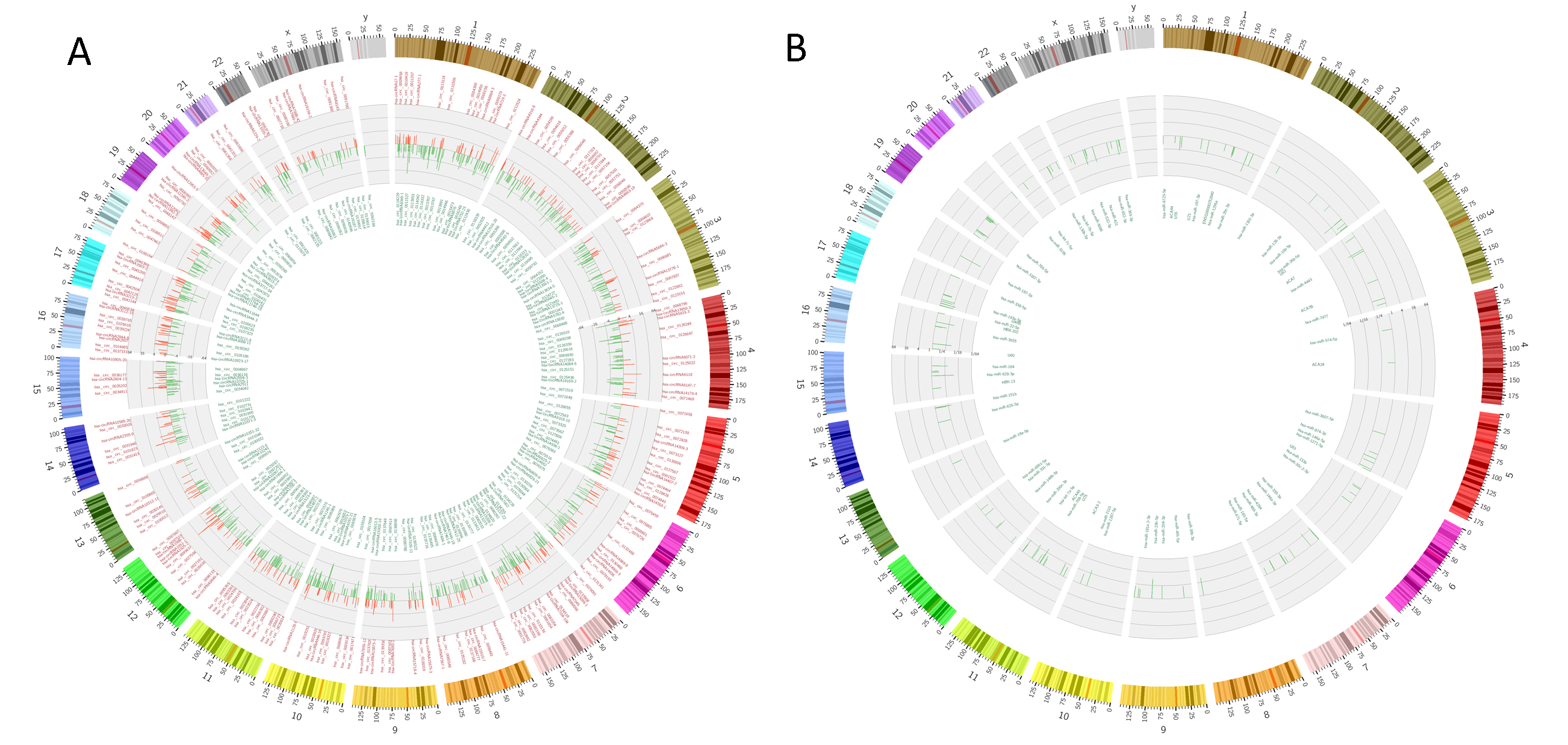

Supplement: Supplementary file 2 [file Image2.TIF]

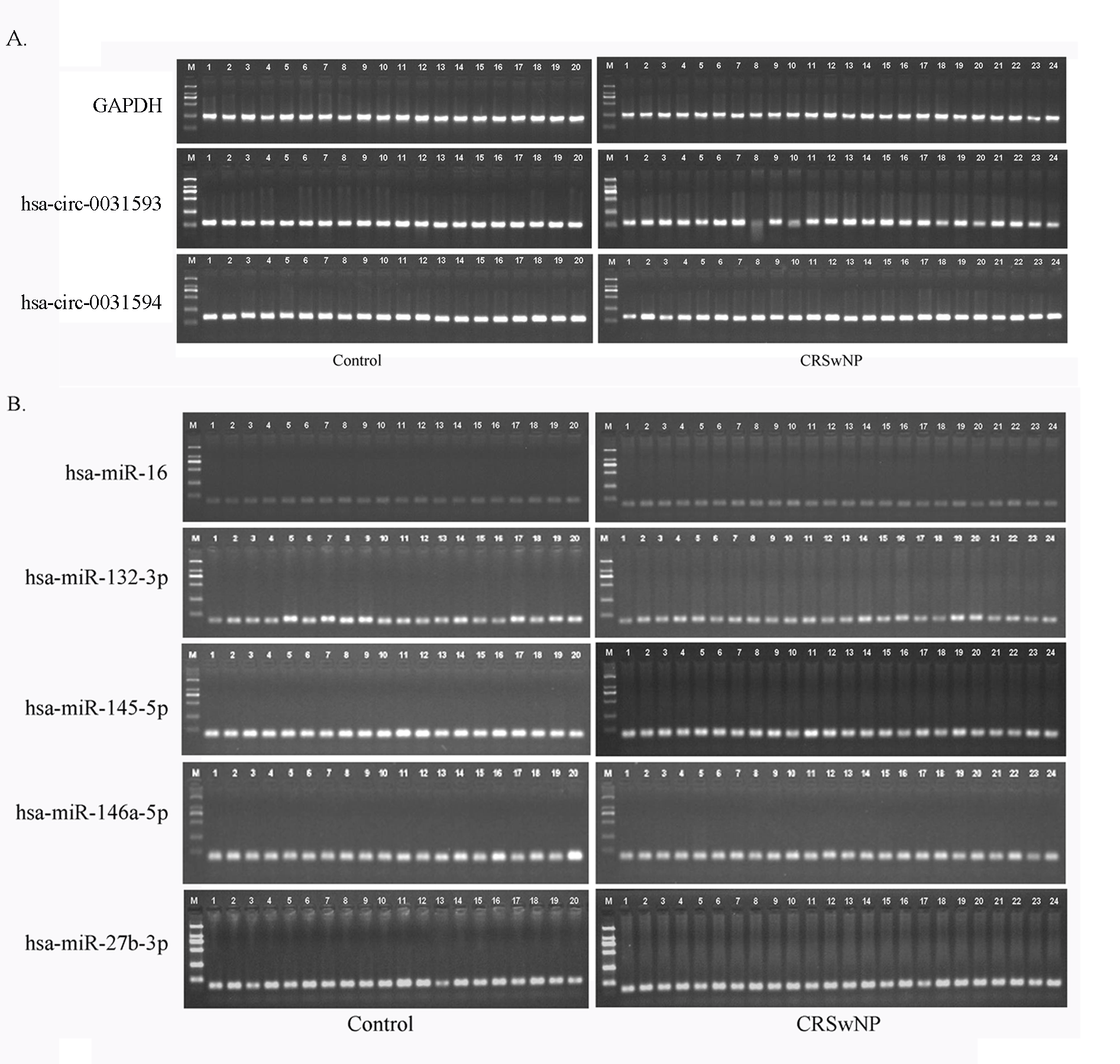

Supplement: Supplementary file 3 [file Image1.TIF]
