# Supplementary material for: Gene Expression Profiles of Circular RNAs and MicroRNAs in Chronic Rhinosinusitis With Nasal Polyps
Source: Front Mol Biosci. 2021 May 28;8:643504. doi: 10.3389/fmolb.2021.643504 (PMC8194396; doi:10.3389/fmolb.2021.643504)
Supplement: Supplementary file 4 [file DataSheet1.docx]

**Supplementary Tables 1. Summary of patient’s characteristics.**

| Parameters | Control | ECRSwNP | non-ECRSwNP |
| --- | --- | --- | --- |
| Methodology used |  |  |  |
| RNA sequencing (1^st^ cohort) | n=3 | n=3 | n=3 |
| Age, yr [mean±SD] | 38±5 | 43±7 | 41±7 |
| Gender [M/F] | 2/1 | 1/2 | 2/1 |
| qRT-PCR(2^nd^ cohord) | n=5 | n=5 | n=5 |
| Age, yr [mean±SD] | 34±13 | 32±11 | 33±16 |
| Gender [M/F] | 2/3 | 3/2 | 3/2 |
| qRT-PCR(3^rd^ cohord) | n=20 | n=24 | - |
| Age, yr [mean±SD] | 41±13 | 43±12 | - |
| Gender [M/F] | 12/8 | 15/9 | - |

**Supplementary Table 2. Nucleotide sequences of circRNAs primers used for RT-qRCR.**

|  | Forward | Reverse |
| --- | --- | --- |
| GAPDH | TGTTGCCATCAATGACCCCTT | CTCCACGACGTACTCAGCG |
| hsa_circ_0031593 | GGTGGGATCCTGCGGATATTT | CGTGTGGGTTCCTACGATCT |
| hsa_circ_0031594 | GCCAAGCTACATGGTGGGAT | GCACTTCGTGTGGGTTCCTA |
| hsa_circ_0109623 | GGGACCGTGACATTCTTTGC | TCTTCGATGACAACCCCAGC |
| hsa_circ_0000736 | GGGGCACAGAGCGGATTGA | CAATGTTGGCGAGGTCGTCTG |

**Supplementary Table 3. Nucleotide sequences of miRNAs primers used for RT-qRCR.**

|  | Stem-loop Reverse Transcription primer | Anti-sense Primer |
| --- | --- | --- |
| hsa-miR-16 | GTCGTATCCAGTGCAGGGTCCGAGGTATTCGCACTGGATACGACCGCCAA | GCTTGTAGCAGCACGTAAATATTG |
| hsa-miR-27b-3p | GTCGTATCCAGTGCAGGGTCCGAGGTATTCGCACTGGATACGACGCAGAA | GCATTTCACAGTGGCTAAGTTC |
| hsa-miR-145-5p | GTCGTATCCAGTGCAGGGTCCGAGGTATTCGCACTGGATACGACAGGGAT | CCAGCAGTCCAGTTTTCCCA |
| hsa-miR-146a-5p | GTCGTATCCAGTGCAGGGTCCGAGGTATTCGCACTGGATACGACAACCCA | CGCATTGAGAACTGAATTCCA |
| hsa-miR-132-3p | GTCGTATCCAGTGCAGGGTCCGAGGTATTCGCACTGGATACGACCGACCA | CGCAATAACAGTCTACAGCCAT |
